# Soluble Immune Checkpoint-Related Proteins in Blood Are Associated With Invasion and Progression in Non-Small Cell Lung Cancer

**DOI:** 10.3389/fimmu.2022.887916

**Published:** 2022-07-06

**Authors:** Qinchuan Wang, Yue He, Wanlu Li, Xiaohang Xu, Qingfeng Hu, Zilong Bian, Andi Xu, Huakang Tu, Ming Wu, Xifeng Wu

**Affiliations:** ^1^ Center for Biostatistics, Bioinformatics and Big Data, The Second Affiliated Hospital and School of Public Health, Zhejiang University School of Medicine, Hangzhou, China; ^2^ Department of Surgical Oncology, Affiliated Sir Run Run Shaw Hospital, Zhejiang University School of Medicine, Hangzhou, China; ^3^ The Key Laboratory of Intelligent Preventive Medicine of Zhejiang Province, Hangzhou, China; ^4^ Department of Thoracic Surgery, Second Affiliated Hospital, Zhejiang University School of Medicine, Hangzhou, China

**Keywords:** soluble immune checkpoint-related protein, non-small cell lung cancer, adenocarcinoma in situ, invasive adenocarcinoma, prediction model

## Abstract

**Background:**

Immune checkpoint inhibition therapy has been achieved significant success in the treatment of non-small cell lung cancer (NSCLC). However, the role of soluble immune checkpoint- related proteins in NSCLC remains obscure.

**Methods:**

We evaluated the circulating levels of 14 immune checkpoint-related proteins panel (BTLA, LAG-3, GITR, IDO, PD-L2, PD-L1, PD-1, HVEM, Tim-3, CD28, CD27, CD80, CD137 and CTLA-4) and their associations with the risk of invasive disease and the risk of NSCLC in 43 pre-invasive (AIS), 81 invasive NSCLC (IAC) patients and matched 35 healthy donors using a multiplex Luminex assay. Gene expression in tumors from TCGA were analyzed to elucidate potential mechanisms. The multivariate logistic regression model was applied in the study. ROC(receiver operator characteristic) curve and calibration curve were used in the performance evaluation.

**Results:**

We found that sCD27, sCD80, CD137 and sPDL2 levels were significantly increased in IAC cases compared to AIS cases (*P*= 1.05E-06, 4.44E-05, 2.30E-05 and 1.16E-06, respectively), whereas sPDL1 and sPDL2 levels were significantly increased in NSCLC cases compared to healthy controls (*P*=3.25E-05 and 1.49E-05, respectively). Unconditional univariate logistic regression analysis indicated that increased sCD27, sCD80, sCD137, and sPDL2 were significantly correlated with the risk of invasive diseases. The model with clinical variables, sCD27 and sPDL2 demonstrated the best performance (AUC=0.845) in predicting the risk of IAC. CD27 and PDCD1LG2 (PDL2) showed significant association with cancer invasion signature in TCGA dataset.

**Conclusion:**

Our study provides evidence that soluble immune checkpoint-related proteins may associate with the risk of IAC, and we further established an optimized multivariate predictive model, which highlights their potential application in the treatment of NSCLC patients. Future studies may apply these biomarkers to test their predictive value of survival and treatment outcome during immunotherapy in NSCLC patients.

## Introduction

Lung cancer is one of the most commonly diagnosed cancers and is also the leading cause of cancer-associated death worldwide, which estimated 2.2 million new incidences and 1.79 million death per year (1). Non-small cell lung cancer (NSCLC) accounts for 85% of lung cancer. Within NSCLC, adenocarcinoma and squamous cell carcinoma are the major subtypes. Immune checkpoint blockade (ICB) has been achieved substantial progression in treatment-naïve NSCLC (2–4). Although ICB therapy has been extensively applied in the treatment of NSCLC, few studies were conducted on the soluble immune checkpoint proteins in the blood, especially in early-stage NCSLC patients.

The evolution from a pre-invasive tumor [adenocarcinoma *in situ* (AIS) or minimally invasive adenocarcinoma (MIA)] to an invasive tumor (invasive adenocarcinoma, IAC) is a milestone event during carcinogenesis of lung adenocarcinoma (LUAD). The outcome of a pre-invasive tumor is excellent if it is detected and resected on time (5). DNA methylation aberrations, aberrant bile acid metabolism, anti-tumor immunity and KRAS, TP53 mutations have been reported associated with increased frequency from AIS to IAC (6–9). Moreover, compared to normal lung tissue and AIS, IAC demonstrated significantly decreased T cell infiltration, more divergent TCR repertoire, increased HLA loss and promoter hypermethylation, which emphasized the substantial role of anti-tumor immunity during NSCLC development (9). However, the biomarkers predicting invasive cancer are still insufficient.

Soluble immune checkpoint-related proteins are mainly derived from tumor cells/immune cells, which have been identified associating with survival, recurrence, and treatment of multiple types of cancer (10). Soluble PD-L1 (sPD-L1) level was reported positively associated with tumor PD-L1 expression at the same timepoint (11). In another study, the sPD-L1 level was correlated with poor prognosis of advanced NSCLC patients (12). sPD-L1 was identified as a negative predictive biomarker of PD-1 inhibitor for its combination with PD-1 in blood (13). Further, Peng et al. reported that soluble LAG3 was significantly elevated in early-stage NSCLC patients, whereas soluble CD27, CD137, TIM-3 and were significantly higher in advanced-stage NSCLC patients (14). Soluble immune checkpoint-related proteins may alter anti-tumor immunity by combining with corresponding immune checkpoint receptor/ligand, thereby affecting outcomes of the patients (10). However, the associations between soluble immune checkpoint-related proteins and lung cancer development remain obscure.

To identify the role of soluble immune checkpoint-related proteins in NSCLC development, we implemented a two-stage study. First, we systematically profiled soluble immune checkpoint-related proteins level in plasma in a cohort of NSCLC patients and matched healthy controls from The Second Affiliated Hospital (SAH), Zhejiang University. Second, we analyzed immune checkpoint gene expression in an external set of NSCLC tumor data (LUAD) from The Cancer Genome Atlas (TCGA), *in silico* functional validation was performed using the data. In short, it is an integrated, multi-stage study involving soluble immune checkpoint-related proteins level, tumoral transcriptomic data from SAH and TCGA.

## Methods and Materials

### Study Population and Data Collection

A schematic design of the study was depicted in **Figure S1**. All the NSCLC patients were recruited from an ongoing NSCLC patient cohort at SAH, Zhejiang University (Hangzhou, Zhejiang) initiated in 2020. The study has been approved by the Institutional Review Board of SAH. The inclusion criteria of the study were as follows: 1. Pathologically confirmed NSCLC. 2. Informed consent or waiver of consent provided by the patient; and follow-up information available. We excluded patients with 1. Non-NSCLC or multiple cancer. 2. Failure to provide informed consent. The healthy controls participated in this study were recruited from an ongoing study on healthy individuals at SAH. All the participants in this study have signed written informed consent before participation.

All the details of clinical-pathological information were obtained from careful chart review. Epidemiological data were collected by SAH staff interviewers through in-person interviews. After interview and consent, a 20 ml blood sample from each participant was collected in up to 4 vacutainer tubes (Fisher Scientific, MA); including 2 lavender top (sodium EDTA), 2 red (no additive) and sent to the laboratory. All patients were not previously untreated by surgery or chemotherapy. Participants were considered as non-smokers if they had smoked less than 100 cigarettes in a lifetime, otherwise they were considered as smokers. The plasma and peripheral mononuclear blood cell (PBMC) were separated and stored in liquid nitrogen for further research. The genomic DNA was extracted from PBMCs using QIAamp 96 DNA QIAcube HT Kit (QIAGEN, USA). All participants of this study were Han Chinese. To reduce the confounding effect, healthy controls, NSCLC patients with cancer *in situ* (AIS), early-stage IAC and late-stage IAC were matched with age, gender using the propensity matching method (PSM) at the ratio of 1.

### Detection of Soluble Immune Checkpoint Proteins in Plasma

Plasma levels of soluble immune checkpoint-related proteins were profiled in duplicate using ProcartaPlex Human Immuno-Oncology Checkpoint Panel (Thermo Fisher, MA) in 96-well plate format to quantify 14 human immune checkpoint markers. The assay was performed according to the protocol provided by the manufacturer employing Bio-plex 200 (Bio-Rad, CA) and Bio-Plex Manager™ 6.0 software. The procedure of protein quantification in plasma is systematically narrated in our previous study (15).

### Statistical Analysis

We applied a logistic regression model to predict the risk of progressing from AIS to IAC with 9 variables and cancer stages of patients. Cutoff points for biomarkers were based on the first quartile value in the sample. Comparison of biomarkers between different stages was carried out using the Wilcoxon rank-sum test. For multiple group comparisons, Bonferroni correction was applied to p-value calculation. Logistic regression analyses were performed, by sequentially adding biomarkers of interest, to identify statistically significant biomarkers associated with increased risk of progressing to the invasive stage in multivariate models. Multicollinearity was tested for models, and all variables have a value of VIF well below 5. Odds ratios and 95% confidence intervals (CI) were estimated for each variable.

Models were developed by adding biomarkers of interest (CEA, CD27, PDL2) sequentially. Modeling started with health history only (model 1: age, gender, BMI, hypertension, smoking), followed by adding single biomarkers of interest (model 2: model 1 + CEA), and then followed by combinations of three biomarkers (model 3: model 1 + sCD27 + sPDL2). A likelihood ratio test was performed to assess the statistical significance of adding biomarkers of interest. Since logistic regression is a probabilistic model, the model assigns probability of progression to IAC to each patient. The decision threshold of 0.6 was selected by optimizing the Brier score. If the predicted probability is great than 0.6, then the patient is labeled as 1(IAC).

The performance of models was evaluated by ROC (receiver operator characteristic) curve, calibration curve and evaluation metrics including area under the ROC curve (AUC), accuracy, sensitivity, specificity, positive predictive value and negative predictive value and Brier score in the test datasets. We randomly split the overall samples into training (80%) and testing (20%) with 1000 iterations, and for each iteration, we trained logistic regression model in the training data, and evaluated the model performance in testing data. The mean and standard deviation of performance metrics were calculated from 1000 evaluations on testing datasets.

Statistical analysis was performed in R (version 4.1.0). Statistical tests for comparison of biomarkers (continuous) between different stages were achieved by R-package rstatix. The *P*-value was provided as a result of a two-tailed test. The logistic regression was applied to calculate OR with R-package stat, and its corresponding 95% CIs were calculated from R-package broom. The ROC curve and AUC analysis were performed using R-package pROC and ggplot2. The calibration curve was obtained using R-package rms. The model performance metrics (accuracy, sensitivity, specificity, positive predictive value, negative predictive value) were obtained from R-package caret and epiR.

## Results

### Patient Characteristics

A total of 159 participants were enrolled in this study, including 43 AIS, 50 early-stage (I,II) IAC, 31 late-stage(III, IV) IAC and 35 controls. The demographic and clinical information were listed in **Table 1**. Among all subjects, the mean age of NSCLC cases and healthy donors were 56.40 and 59.89 years, respectively. Over half of cases (58.1%) were females, whereas 28.6% of healthy donors were females. Over a third of cases (36.7%) and 48.6% of healthy donors were smokers.

**Table 1:** Distribution of soluble immune checkpoint-related proteins in control, AIS and IAC patients.

	Control (N=35)	AIS (N=43)	IAC (N=81)						
			Early stage (N=50)	Late stage (N=31)					
Marker	Median (IQR) pg/ml	Median (IQR) pg/ml	Median (IQR) pg/ml	Median (IQR) pg/ml	*P	**P	***P	****P	†*P trend*
sBTLA	280.52 (113.59-511.51)	228.01 (181.84-274.19)	211.52 (170.17-290.44)	244.28 (178.41-329.29)	0.68	0.55	0.98	0.25	0.98
sCD27	556.25 (466.54-681.61)	489.69 (399.67-629.86)	735.4 (476.1-1198.23)	1382.75 (943.44-1745.74)	**2.24E-01**	**5.37E-05**	**1.05E-06**	**2.93E-04**	**7.80E-09**
sCD28	64.51 (21.04-161.75)	31.77 (22.24-44.18)	29.67 (23.31-38.53)	49.74 (32.81-71.38)	0.19	0.29	0.73	0.14	0.84
sCD80	106.43 (67.27-137.99)	66.42 (43.94-91.73)	109.73 (67.42-341.77)	285.04 (90.3-398.26)	**1.46E-02**	**9.74E-02**	**4.44E-05**	**0.03**	**5.00E-04**
sCD137	177.01 (149.97-257.58)	65.11 (46.03-111.6)	132.06 (79.18-188.46)	176.76 (130.55-216.77)	**2.85E-05**	**4.59E-02**	**2.30E-05**	**0.02**	0.19
sCTLA4	7.81 (6.74-20.85)	5.19 (2.46-6.83)	5.91 (3.89-9.37)	9.63 (5.35-10.64)	**0.03**	0.30	0.14	0.25	0.93
sGITR	37.98 (16.02-68.48)	18.1 (9.7-71.37)	10.64 (6.92-26.63)	10.64 (6.85-17.99)	0.87	0.08	0.16	0.67	**0.04**
sHVEM	13.86 (8.98-22.35)	4.87 (2.59-17.23)	20.08 (14.71-29.34)	22.51 (16.71-41.88)	0.08	**0.01**	**0.02**	0.6	**0.01**
sIDO	6.68 (4.52-10.7)	5.89 (4.15-8.21)	6.88 (4.15-12.42)	8.96 (3.62-14.79)	0.37	0.76	0.2	0.64	0.51
sLAG3	12.97 (9.93-16.02)	15.09 (15.09-15.09)	14.63 (9.31-20.24)	10.66 (6.4-17.47)	1.00	0.93	–	0.33	0.37
sPD1	13.78 (9.35-24)	12.39 (7.2-16.06)	16.16 (11.23-22.43)	17.05 (12.01-20.95)	0.36	0.64	**0.01**	0.46	0.08
sPDL1	0.35 (0.32-0.45)	0.29 (0.24-0.37)	0.18 (0.11-0.29)	0.24 (0.13-0.29)	**3.82E-03**	**3.63E-05**	0.05	0.87	**3.88E-05**
sPDL2	268.82 (218.56-339.59)	321.21 (228.13-402.55)	467.19 (349.75-694.05)	702.54 (388.59-852.24)	0.21	**3.65E-08**	**1.16E-06**	0.07	**1.99E-10**
sTIM3	131.31 (113.25-155.83)	28.98 (17.84-56.54)	39.49 (16.45-66.41)	67.75 (28.78-156.22)	**2.347E-03**	**0.04**	0.33	0.11	0.96

AIS, adenocarcinoma in situ; IAC, invasive adenocarcinoma. Early stage indicates stage I&II disease, Late stage indicate stage III&IV disease, the staging criteria according to NCCN Clinical Practice Guidelines Non-small cell lung cancer v1, 2022 *P indicates Control vs. AIS, **P indicates Control vs. IAC, ***P indicates AIS vs. IAC, ****P indicates early stage vs. late stage, †P for trend was analyzed using Jonckheere-Terpstra test.

### Soluble Immune Checkpoint-Related Proteins Were Associated With Lung Cancer Invasion

The Luminex multiplex assay was performed evaluating all soluble immune checkpoints in plasma on all participants (**Table S1**). Soluble LAG3, HVEM, GITR, CTLA4, PDL1 and TIM3 were not included in the subsequent analysis for minimal variation among samples or too many missing values.

**Figure 1:**
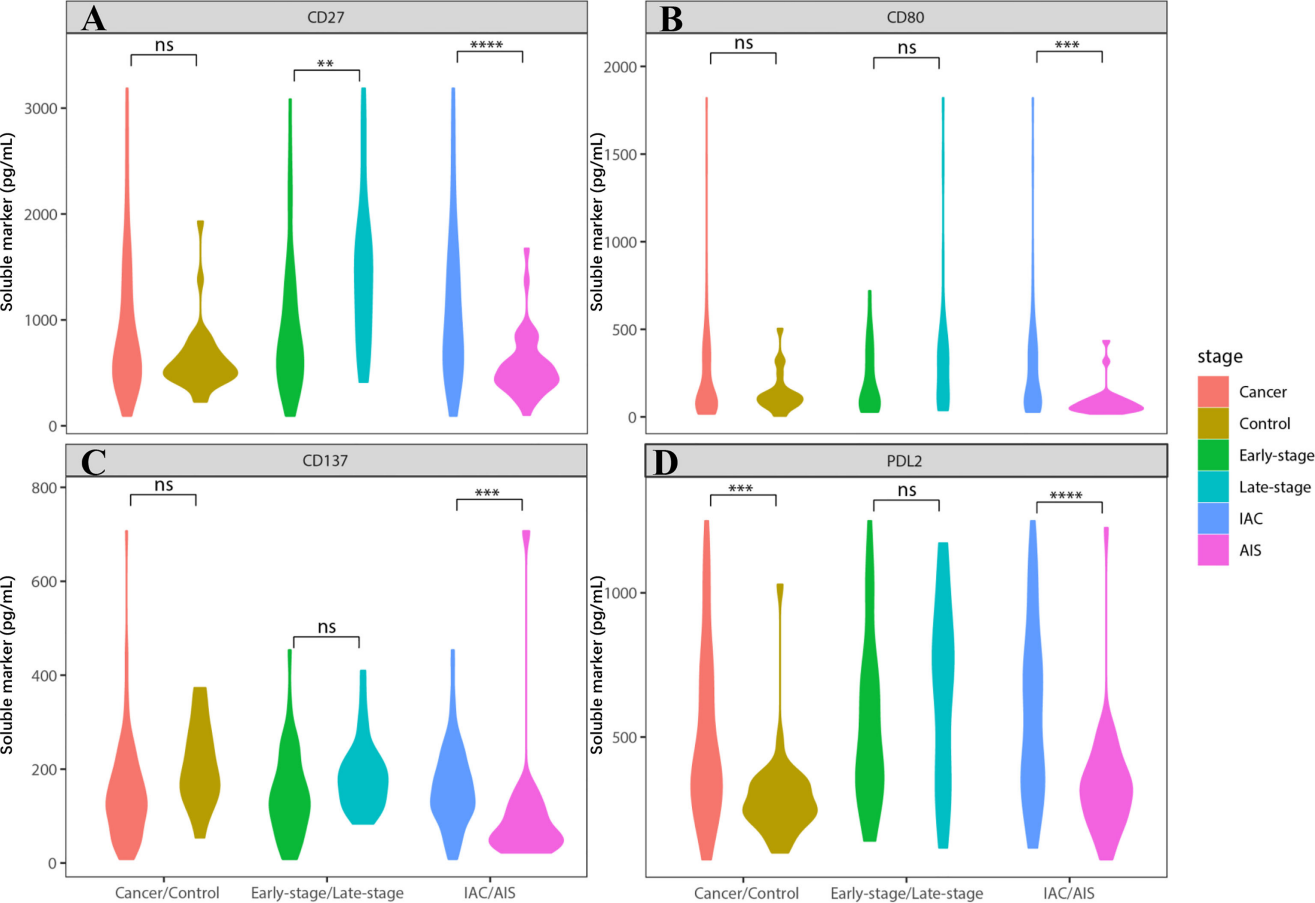
Soluble immune checkpoint-related proteins were associated with lung cancer invasion. Soluble immune checkpoint-related proteins level (sCD27, sCD80, sCD137 and sPDL2) were significantly increased in IAC patients. **(A)** sCD27 level was significantly elevated in IAC patients (vs. AIS patients) (*P*=1.05E-06). It was also increased in late-stage IAC patients (vs. early-stage) (*P*=2.93E-04). **(B)** sCD80 level was significantly elevated in IAC patients, compared to AIS patients (*P*=4.44E-05). **(C)** sCD137 level was significantly elevated in IAC patients, compared to AIS patients (*P*=2.30E-05). It was also increased in late-stage IAC patients (vs. early-stage) (*P*=0.02), and controls (vs. cancer) (*P*=0.01). However, the data in late-stage and control is not conceivable due to too many missing values. **(D)** sPDL2 level was significantly elevated in IAC patients (vs. AIS patients) (*P*=1.16E-06). It was also increased in controls (vs. cancer) (*P*=1.49E-05). ** indicates *P* value < 0.01, *** indicates *P* value < 0.001, **** indicates *P* value < 0.0001. NS indicates not significant.

We found that sCD27, sCD80, and sPDL2 levels were significantly increased in IAC cases compared to AIS cases (**Figure 1**; **Table S1**, *P*= 1.05E-06, 4.44E-05, and 1.16E-06, respectively), whereas sPDL1 and sPDL2 levels were significantly increased in NSCLC cases compared to controls (*P*=3.25E-05 and 1.49E-05, respectively). Unconditional univariate logistic regression analysis indicated that increased soluble CD27, CD80, CD137, and PDL2 were significantly associated with the risk of invasive diseases (**Table S2**). Pre-treatment level of CEA was also analyzed, which demonstrated that it was significantly elevated in late-stage (*P*<0.05, vs. early stage) and IAC (*P*<0.001, vs. AIS) NSCLC cases (**Figure S2**).

### Risk Modeling of Invasive Disease

We performed multi-variate logistic regression models to predict the invasion of NSCLC (**Table 2**). The discriminatory accuracy, which measures how well a prediction model distinguishes at the individual level between those who will develop invasive disease and those who will not, was assessed by OR and AUC values in three models. In model 1 (clinical variables only), smoking (smokers vs non-smokers) was significantly associated with increased risk of IAC (OR=9.72, 95% CI = 2.00-59.10). In model 2 (clinical variables and CEA), increasing levels of CEA (>1.44 ng/mL) were not associated with IAC risk. In model 3 (clinical variables, sCD27 and sPDL2), high levels of sPDL2(above 287 pg/mL) remained significant in predicting IAC risk (OR = 4.23, 95% CI = 1.20-17.70), increasing levels of sCD27 (above 453 pg/mL) were also associated with IAC risk (OR = 2.9, 95% CI = 0.94-9.44), but not statistically significant.

**Table 2:** Different models of multi-variate logistic regression in prediction of invasive disease.

Characteristics	Model1	Model2	Model3
	OR (95% CI)	OR (95% CI)	OR (95% CI)
Age			
<=60	1 (reference)	1 (reference)	1 (reference)
>60	1.63 (0.63-4.32)	1.41 (0.48-4.22)	1.15 (0.4-3.32)
Gender			
Female	1 (reference)	1 (reference)	1 (reference)
Male	1.35 (0.35-5.26)	1.36 (0.3-6.25)	3.1 (0.64-17.06)
BMI			
<=25	1 (reference)	1 (reference)	1 (reference)
>25	1.6 (0.6-4.46)	1.52 (0.51-4.71)	1.59 (0.55-4.78)
Hypertension			
No	1 (reference)	1 (reference)	1 (reference)
Yes	2.21 (0.75-7.13)	2.91 (0.85-11.87)	2.81 (0.85-10.66)
Smoking			
No	1 (reference)	1 (reference)	1 (reference)
Yes	9.72 (2-59.1)	8.29 (1.53-55.2)	6.68 (1.23-45.19)
CEA			
Low	—	1 (reference)	—
High	—	1.88 (0.69-5.18)	—
sCD27			
Low	—	—	1 (reference)
High	—	—	2.9 (0.94-9.44)
sPDL2			
Low	—	—	1 (reference)
High	—	—	4.23 (1.2-17.7)

Model1: epidemiology variables, Model 2: epidemiology variables + CEA, Model 3: epidemiology variables + sCD27 + sPDL2.

**Figure 2:**
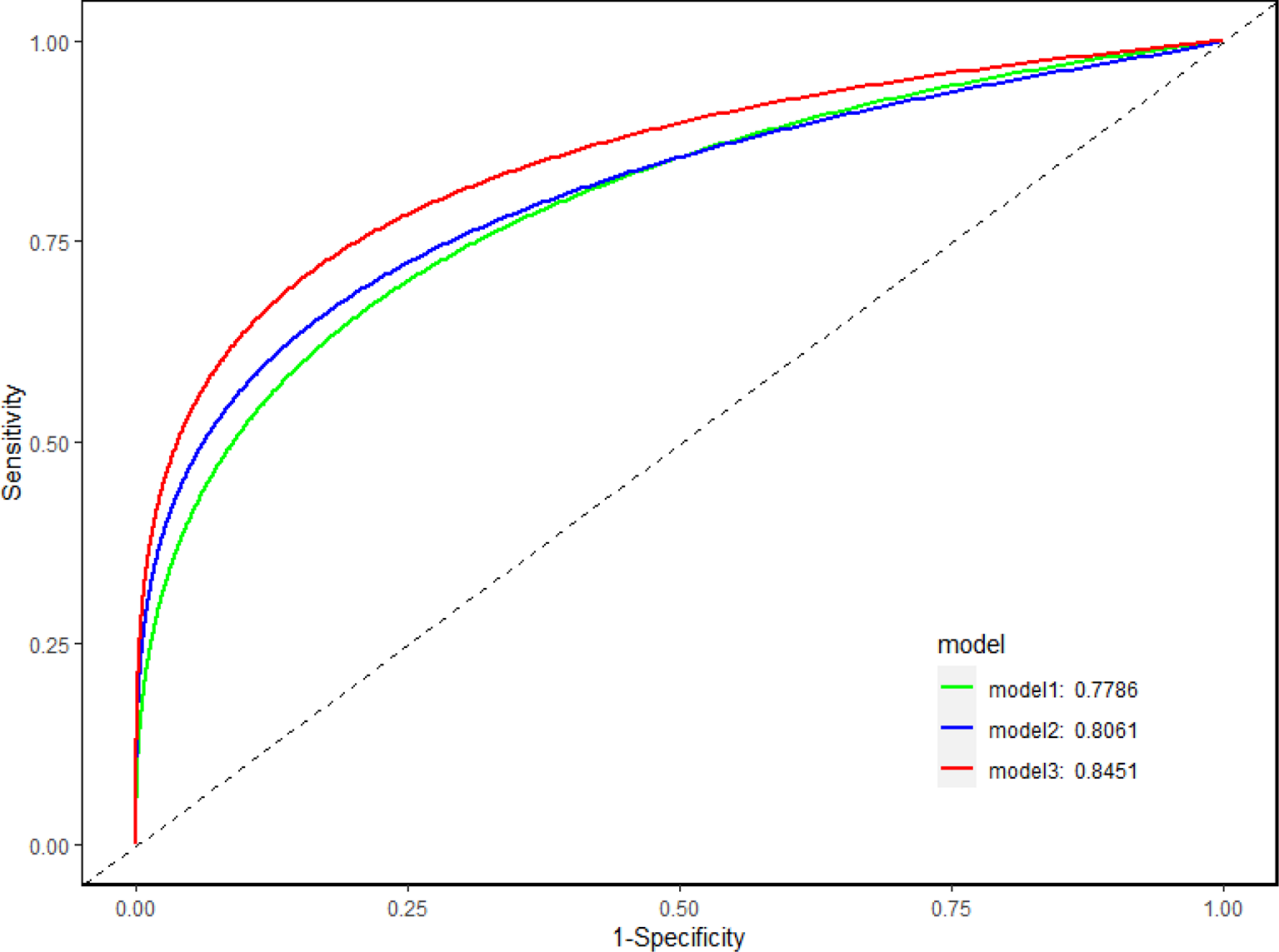
Discriminatory accuracy of the models. Discriminatory accuracy for predicting the risk of IAC was assessed by constructing receiver operating characteristic (ROC) curves and calculating the area under the curve (AUC). In model 1, the AUC value was 0.789 based on clinical variables only (age, gender, BMI, hypertension, smoking) data. The AUC was increased to 0.806 in model 2 with CEA, and 0.845 in model 3 with sCD27 and sPDL2.

AUC was applied to illustrate the changes in model discriminatory accuracy by comparing different models (**Figure 2**; **Table 3**). In model 1, the AUC value was 0.789 based on clinical variables only data. The AUC was increased to 0.806 in model 2 with CEA, and 0.845 in model 3 with sCD27 and sPDL2.

**Table 3:** Performance measures of prediction models.

Performance Measures	Model 1 (95%CI)	Model 2 (95%CI)	Model 3 (95%CI)
Accuracy	0.718 (0.63-0.795)	0.723 (0.631-0.804)	0.774 (0.69-0.844)
AUC	0.779 (0.699-0.858)	0.806 (0.727-0.885)	0.845 (0.779-0.911)
Sensitivity	0.814 (0.666-0.916)	0.75 (0.578-0.879)	0.814 (0.666-0.916)
Specificity	0.667 (0.553-0.768)	0.711 (0.595-0.809)	0.753 (0.645-0.842)
Positive Predictive Value	0.565 (0.433-0.69)	0.551 (0.402-0.693)	0.636 (0.496-0.762)
Negative Predictive Value	0.871 (0.761-0.943)	0.857 (0.746-0.933)	0.884 (0.784-0.949)
Brier Score	0.174	0.164	0.152

**Figure 3:**
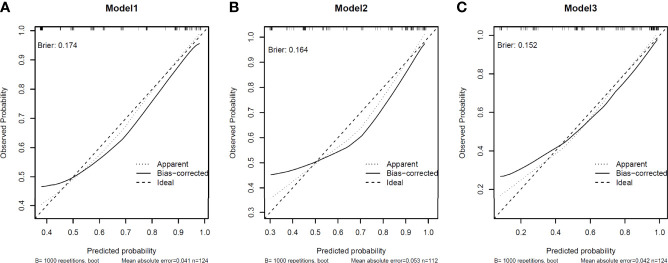
Calibration of the models. Model calibration was assessed by in-sample calibration and bias-corrected calibration with 1000 bootstrap resampling procedures. Brier score was applied as the index of model calibration. **(A)** In model 1, clinical variables only model displayed brier scores of 0.174. **(B)** In model 2, clinical variables + CEA model displayed brier scores of 0.164. **(C)** clinical variables + sPDL2 and sCD27 displayed brier scores of 0.152.

Model calibration was assessed by in-sample calibration and bias-corrected calibration with 1000 bootstrap resampling procedures. The calibration curve for all models was depicted in **Figure 3**, showing that models with sPDL2 and sCD27 as covariate (model 3) displayed smaller Brier scores (0.152) compared to models without sPDL2 and sCD27 (model 1, brier score=0.174; model 2, brier score=0.164). In addition, models with sPDL2 and sCD27 as covariate (model 3) are better calibrated compared to models without sPDL2 and sCD27 based on smaller mean absolute error and smaller gap between the biased-corrected line and the idea line. For all models, there is some underestimation at lower predicted probabilities.

### Immune Checkpoint Associated Genes Expressions Were Associated With Cancer Invasion Signature

**Figure 4:**
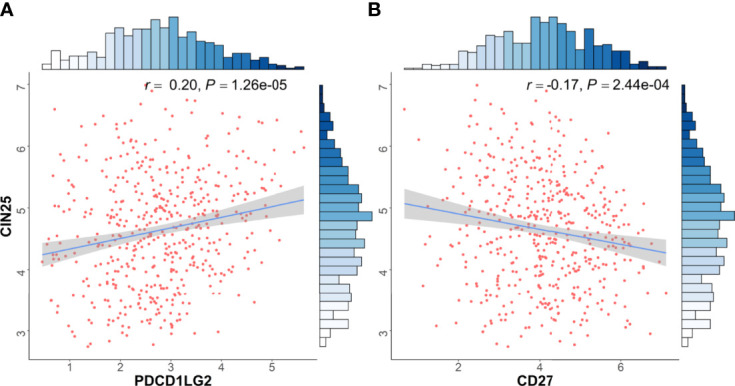
Immune checkpoint associated genes expressions were associated with cancer invasion signature. Transcriptomic data from TCGA dataset was used in functional exploration. CIN25 score was applied as signature of cancer invasion according to previous literatures [15, 16]. **(A)** CD27 expression was negatively associated with CIN25 score (rho=-0.17, *P*=2.44E-04), **(B)** PDL2 expression was positively associated with CIN25 score (rho=0.20, *P*=1.26E-05).

To further explore the mechanism underlying CD27 and PDL2 with NSCLC invasion, we identified the association between CD27/PDL2 expression and cancer invasion signature (CIN25) (16, 17) in LUAD from TCGA database. The results showed that CD27 expression was negatively associated with CIN25 score (rho=-0.17, *P*=2.44E-04), whereas PDL2 expression was positively associated with CIN25 score (rho=0.20, *P*=1.26E-05) (**Figure 4**).

## Discussion

To date, published studies only reported associations between soluble immune checkpoint-related protein levels and early detection of NSCLC. In this study, we systematically profiled soluble immune checkpoint protein levels in matched AIS, IAC and healthy controls, and we developed prediction models based on clinical variables and soluble immune checkpoint proteins to discriminate AIS from IAC patients. Our results demonstrated that sCD27 levels were significantly associated with the development of NSCLC, which range from healthy donors, AIS, early-stage and late-stage IAC. Further, we identified the model with sCD27 and sPDL2 was best able to predict the risk of IAC, even better than the model with CEA. The functional validation using TCGA data indicated that CD27 and PDL2 expression is significantly associated with cancer invasion signature, which illustrated potential mechanisms. Therefore, our findings elaborated the significance of soluble immune checkpoint proteins in the prediction of invasive NSCLC, and further explored the possible mechanisms.

CD27 is a promising target of immunotherapy. CD27 is a stimulatory immune checkpoint mainly found on the surface of immune cells, which is the receptor of CD70 (18). Soluble CD27 is a 32kDa cleaved isoform of membrane-bound CD27, which has been reported to interfere interaction of CD27 and CD70 (19). In our study, sCD27 was significantly associated with invasive disease in NSCLC patients, and it was also an ideal predictor of cancer invasion, outperforming CEA. This finding is consistent with a previous study indicating that sCD27 is associated with the cancer stage of IAC in NSCLC patients (14), however, no AIS was included in this study. Moreover, a high sCD27 level was associated with poor performance and reduced survival in the advanced stage of NSCLC patients (20). Another study reported that high serum sCD27 level was correlated with inferior prognosis in acute myeloid leukemia patients (21). sCD27 might interfere with the conjugation of membrane-bound CD27 and CD70, thereby affecting the priming of CD8+ T cells and subsequent anti-tumor immunity. This explains the potential mechanism of our findings. Furthermore, we identified that CD27 expression is negatively associated with cancer invasion signature (CIN25), indicating enforced CD27 expression in tumors may inhibit cancer invasion. CD27 agonists could promote human αβ and γδ T cells proliferation, cytokine production, and CD8+ T cell activation (22, 23). These literature supported our finding on CD27 in LUAD. Anti-CD27 mAb, varlilumab could enhance T cells expansion and reduce the frequency of Tregs in the PBMC of cancer patients (24, 25). Therefore, targeting CD27 through reducing sCD27 production and agonizing CD27 may decrease the risk of developing invasive disease and improve the prognosis in NSCLC patients, though further validation is warranted.

PDL2 is one ligand of PD1, whose functions in carcinogenesis are not clear. PDL2 expressed on tumor cells may have an inhibitory effect on effector T cells through modulation of Th2 responses (26, 27). sPDL2 is mainly thought to be produced by cleavage of membrane-bound PDL2, similar to its cousin PDL1 (28). In this study, we identified that a high sPDL2 level was associated with the risk of IAC, and it could help predict invasive disease in treatment-naïve NSCLC patients. This is supported by a recent study indicating that sPDL2 was significantly elevated in NSCLC patients compared to healthy donors, though no AIS patients were included (14). It may be caused by the binding of membrane PD1 on the effector cells, which subsequently promote the immune evasion of tumor cells (27). Also, a low sPDL2 level was reported associated with grade 3/4 toxicities of nivolumab treatment in advanced stage NSCLC patients, indicating its role in immunotherapy (29). sPDL2 was associated with platinum resistance in advanced epithelial ovary carcinoma (30). Furthermore, we identified that PDL2 expression was positively associated with CIN25 signature, which indicated that PDL2 may promote the invasion of NSCLC cells. This is consistent with a previous study showed that high PDL2 expression was associated with smoking and vascular invasion in NSCLC patients (31). Another study also reported that PDL2 expression was associated with lymphatic invasion in lung squamous cell carcinoma (32). Therefore, sPDL2 may promote cancer invasion *via* interacting with membrane PD-1 on immune cells, and high PDL2 expression in the tumor may also increase the risk of invasive disease in NSCLC patients.

Biomarkers and predictive models discriminating AIS and IAC in NSCLC patients are scarce. In this study, we established multi-variate predictive models to predict invasive disease. Models involved sCD27 and sPDL2 showed better efficacy than the model with CEA, which is a classic biomarker associated with cancer progression. This may be partially caused by the reduced anti-tumor immunity associated with soluble immune checkpoint-related proteins (9). Though other factors including DNA methylation, metabolomics and transcriptomics could affect the progression from AIS to IAC (6, 7, 16), soluble immune checkpoint-related proteins have the strengths of minimally invasive, low-cost and rapid detection, which have significant potential in the era of liquid biopsy. Furthermore, the model developed in this study could help predicting the progression of invasive disease in clinical setting, which could help improving the outcomes of the NSCLC patients, though independent validation is still warranted.

Our study has several strengths including the multiplex profiling of soluble immune checkpoint-related proteins, corresponding immune gene expressions in tumors, and the association analysis of immune gene expression and cancer invasion signature to provide biological validity. We also established and optimized multivariate predictive models of invasive disease, which demonstrate ideal discriminative capability. However, we also acknowledge several limitations. First, we have a limited sample size with few AIS and IAC patients, which might constrain the predictive power of our study. Though the different groups of participants were well designed and matched to prevent potential confounding factors, additional validation within a larger population and independent cohort are necessary. Second, we did not perform mechanistic studies to explore the functional impact of soluble immune checkpoint-related proteins. Instead, we evaluated the associations between identified gene expression and cancer invasion signature to decipher possible mechanisms employing TCGA dataset. Third, we did not evaluate immune checkpoint protein level in peripheral blood mononuclear cells, which could be informative to illustrate the interaction between soluble immune checkpoint-related proteins and membrane-bound immune checkpoints on immune cells. Fourth, this is a retrospective study instead of a prospective study, which may limit the clinical application of our findings, a prospective validation is warranted.

In this study, we identified a panel of soluble immune checkpoint-related proteins associated with the risk of IAC, and we further established an optimized multi-variate predictive model based on our findings. Patients with high levels of sCD27 or sPDL2 may have higher risks of invasive diseases, which require intensified surveillance or treatment. Future studies may apply these markers to test their predictive value of survival and treatment outcome during immunotherapy.

## Data Availability Statement

The original contributions presented in the study are included in the article/**Supplementary Material**. Further inquiries can be directed to the corresponding author.

## Ethics Statement

The studies involving human participants were reviewed and approved by The Second Affiliated Hospital, Zhejiang University School of Medicine. The patients/participants provided their written informed consent to participate in this study.

## Author Contributions

XW involved in conception and design. QW and YH involved in development of methodology. MW, XX, and WL participated in acquisition of data (acquired and managed patients, provided facilities, etc.). HT, YH, QH, WL, and ZB involved in analysis and interpretation of data (e.g., statistical analysis, biostatistics, computational analysis). QW, YH, HT, and XW drafted the manuscript and all the authors participant in the revision of the manuscript. XW involved in study supervision. All authors contributed to the article and approved the submitted version.

## Funding

This work was funded by Key Laboratory of Intelligent Preventive Medicine of Zhejiang Province (2020E10004), Leading Innovative and Entrepreneur Team Introduction Program of Zhejiang (2019R01007) and Key Research and Development Program of Zhejiang Province (2020C03002), Nature and Science Fund Public Program of Zhejiang Province (LGF22H160008), respectively.

## Conflict of Interest

The authors declare that the research was conducted in the absence of any commercial or financial relationships that could be construed as a potential conflict of interest.

## Publisher’s Note

All claims expressed in this article are solely those of the authors and do not necessarily represent those of their affiliated organizations, or those of the publisher, the editors and the reviewers. Any product that may be evaluated in this article, or claim that may be made by its manufacturer, is not guaranteed or endorsed by the publisher.
