# Supplementary material for: Soluble Immune Checkpoint-Related Proteins in Blood Are Associated With Invasion and Progression in Non-Small Cell Lung Cancer
Source: Front Immunol. 2022 Jul 6;13:887916. doi: 10.3389/fimmu.2022.887916 (PMC9296827; doi:10.3389/fimmu.2022.887916)
Supplement: Supplementary file 1 [file DataSheet_1.docx]

Supplementary Material

# Supplementary Figures and Tables

## Supplementary Figures


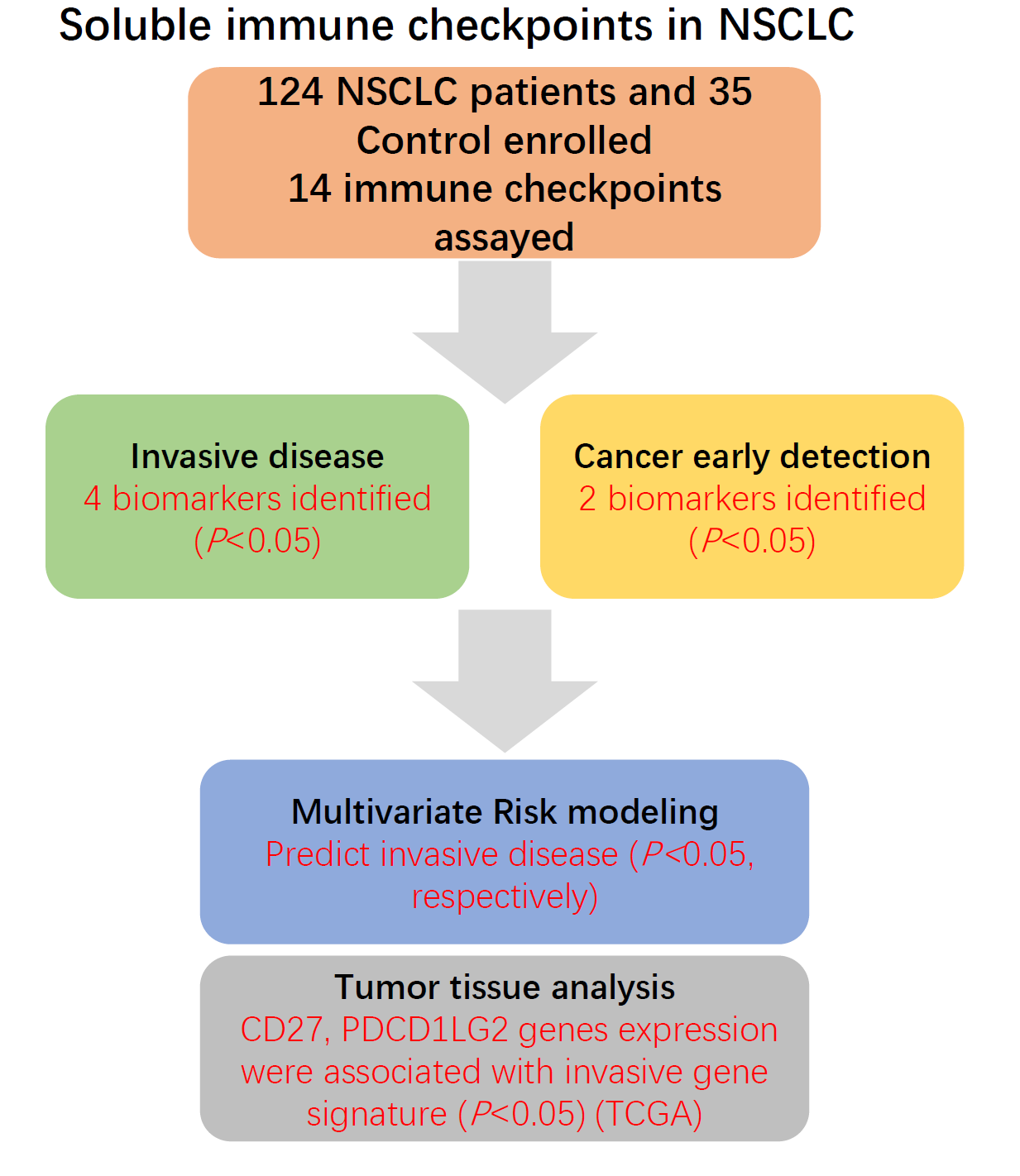


**Supplementary Figure S1.** A schematic design of the study.


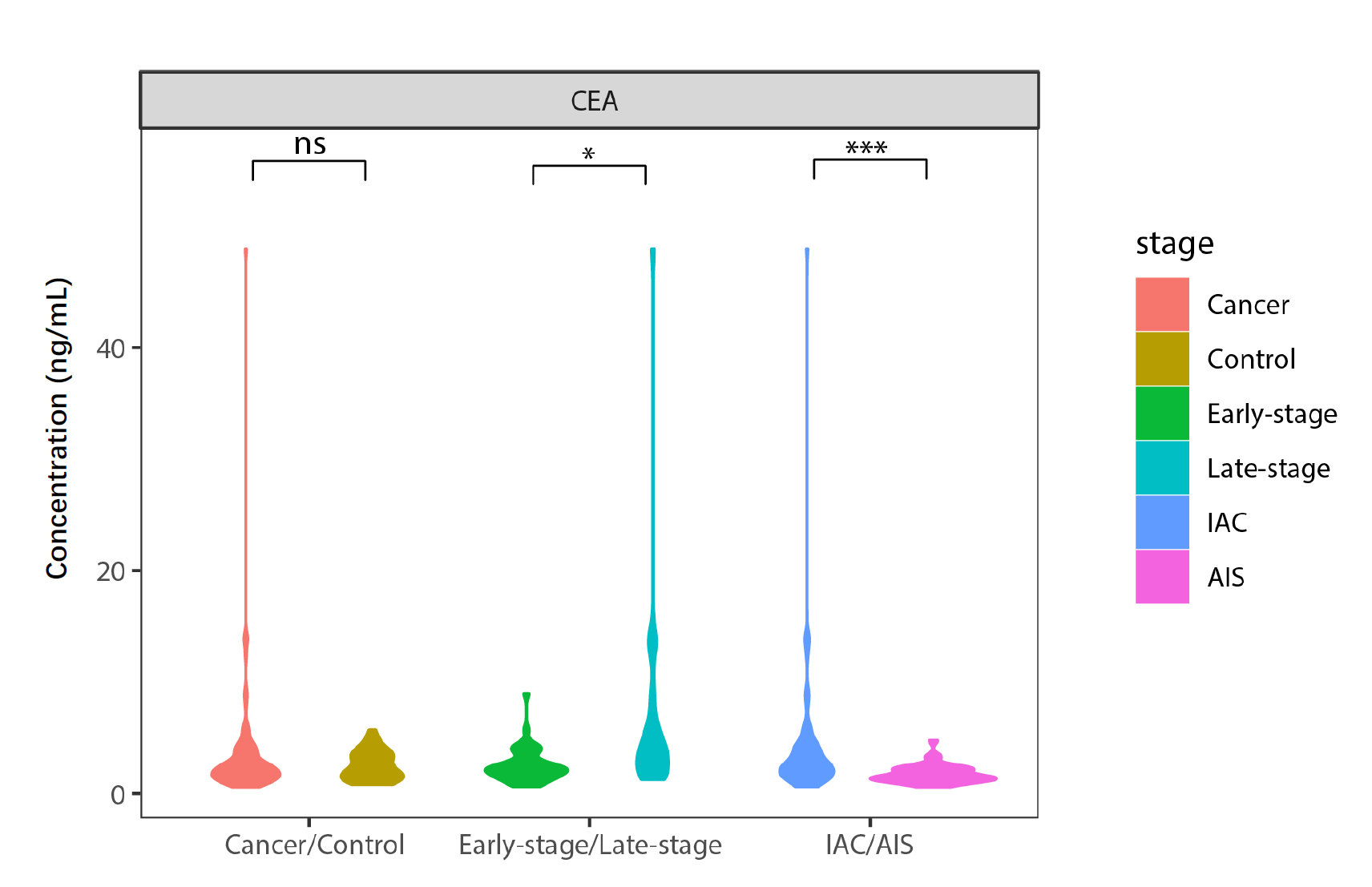


**Supplementary Figure S2:** CEA level was significantly elevated in IAC patients (vs. AIS patients) (*P*<0.001). It was also increased in late-stage IAC patients (vs. early-stage) (*P*<0.05). AIS: Adenocarcinoma in situ, IAC: Invasive adenocarcinoma

## Supplementary Tables

| **Supplemental Table S1. Host Characteristic information of all participants** | | | |  |
| --- | --- | --- | --- | --- |
| Variables | | NSCLC cases, *n* (%) | | Control, *n* (%) |
|  |  | AIS | IAC |  |
| n | | 43 (27) | 81 (50.94) | 35 (22) |
| Age, mean (SD) | | 52.72 (10.62) | 58.36 (10.65) | 59.89 (9.91) |
| BMI, mean (SD) | | 23.31 (2.58) | 24.03 (3.34) | 23.31 (2.51) |
| Sex | |  |  |  |
|  | Male | 7 (16.28) | 45 (55.56) | 25 (71.43) |
|  | Female | 36 (83.72) | 36 (44.44) | 10 (28.57) |
| Age | |  |  |  |
|  | <=60 | 32 (74.42) | 43 (53.09) | 15 (42.86) |
|  | >60 | 11 (25.58) | 38 (46.91) | 20 (57.14) |
| Smoking status | |  |  |  |
|  | No | 40 (93.02) | 40 (49.38) | 18 (51.43) |
|  | Yes | 3 (6.98) | 41 (50.62) | 17 (48.57) |
| BMI | |  |  |  |
|  | <25 | 34 (79.07) | 53 (65.43) | 25 (71.43) |
|  | >=25 | 9 (20.93) | 28 (34.57) | 10 (28.57) |
| Hypertension | |  |  |  |
|  | Yes | 6 (13.95) | 27 (33.33) | 16 (45.71) |
|  | No | 37 (86.05) | 54 (66.67) | 19 (54.29) |
| Stage | |  |  |  |
|  | *Early | - | 50 (61.73) | - |
|  | *Late | - | 31 (38.27) | - |
| Abbreviations: AIS: adenocarcinoma in situ, IAC: invasive adenocarcinoma. * Early stage indicates stage I&II disease, Late stage indicate stage III&IV disease, the staging criteria according to NCCN Clinical Practice Guidelines Non-small cell lung cancer v1, 2022 | | | | |

| **Supplemental Table S2. Univariate logistic regression model of invasive disease of NSCLC** | |
| --- | --- |
| Variables | OR (95%CI) |
| CD80 |  |
| Low | 1 |
| High | 4.44(1.73-11.69) |
| CEA |  |
| Low | 1 |
| High | 3.16(1.32-7.74) |
| IDO |  |
| Low | 1 |
| High | 0.83(0.31-2.20) |
| PD1 |  |
| Low | 1 |
| High | 2.35(0.89-6.11) |
| PDL2 |  |
| Low | 1 |
| High | 3.13(1.36-7.36) |
| BLTA |  |
| Low | 1 |
| High | 0.76(0.25-2.05) |
| CD27 |  |
| Low | 1 |
| High | 4.55(1.96-11.01) |
| CD137 |  |
| Low | 1 |
| High | 7.82(2.88-22.40) |
